# Exogenous Bioactive Peptides Have a Potential Therapeutic Role in Delaying Aging in Rodent Models

**DOI:** 10.3390/ijms23031421

**Published:** 2022-01-26

**Authors:** Jianqiang Wang, Yixin Wu, Zhongxu Chen, Yajuan Chen, Qinlu Lin, Ying Liang

**Affiliations:** Molecular Nutrition Branch, National Engineering Research Center of Rice and By-Product Deep Processing, College of Food Science and Engineering, Central South University of Forestry and Technology, Changsha 410004, China; 20201100385@csuft.edu.cn (J.W.); 20191200410@csuft.edu.cn (Y.W.); 20191100352@csuft.edu.cn (Z.C.); 20201100406@csuft.edu.cn (Y.C.); linqinlu@hotmail.com (Q.L.)

**Keywords:** bioactive peptide, anti-aging, rodents

## Abstract

In recent years, some exogenous bioactive peptides have been shown to have promising anti-aging effects. These exogenous peptides may have a mechanism similar to endogenous peptides, and some can even regulate the release of endogenous active peptides and play a synergistic role with endogenous active peptides. Most aging studies use rodents that are easy to maintain in the laboratory and have relatively homogenous genotypes. Moreover, many of the anti-aging studies using bioactive peptides in rodent models only focus on the activity of single endogenous or exogenous active peptides, while the regulatory effects of exogenous active peptides on endogenous active peptides remain largely under-investigated. Furthermore, the anti-aging activity studies only focus on the effects of these bioactive peptides in individual organs or systems. However, the pathological changes of one organ can usually lead to multi-organ complications. Some anti-aging bioactive peptides could be used for rescuing the multi-organ damage associated with aging. In this paper, we review recent reports on the anti-aging effects of bioactive peptides in rodents and summarize the mechanism of action for these peptides, as well as discuss the regulation of exogenous active peptides on endogenous active peptides.

## 1. Introduction

In modern society, the extension of average life expectancy and the decreased birth rate have led to aging-related burdens across many regions [1,2]. Aging is a dynamic process associated with accumulated cell damage, a decline in biological function, and susceptibility to disease occurring over time [3]. A common and widely recognized mechanism for aging is oxidative damage caused by the accumulation of reactive oxygen species (ROS) [4], resulting from decreased antioxidant capacity, mitochondrial dysfunction, inflammation, etc. [5]. Aging can lead to multiple age-related diseases (ARDs) [6], such as cancer, Alzheimer’s disease (AD), cardiovascular disease (CVD), metabolic syndrome, obesity, fatty liver, and many other chronic diseases. The aging process inevitably involves the aging of cells, which is usually caused by damage at the molecular and cellular level by long-term exposure to endogenous and exogenous stressors. These damaged cells eventually lose their proliferative capacity and promote aging at an organism level [7]. These senescent cells can release a variety of pro-inflammatory factors and chemokines to promote cellular dysfunction, causing senescence-related diseases. In the process of skin aging, oxidative stress and inflammation can increase the activity of matrix metalloproteinases (MMPs) and increase the degradation of collagen, resulting in skin sagging and wrinkle formation. In some neurodegenerative diseases, such as AD, oxidative stress and inflammation can increase the accumulation of amyloid plaques (Aβ) and promote lesions in the brain. Oxidative stress and inflammation also play an important role in the aging of several other organs, such as the heart, liver, and kidneys. Collectively, these pathological changes can cause a variety of complications that affect multiple systems in the body. Thus, ARDs seriously impact the quality of life, shorten the lifespan, and bring a heavy burden to families and society. Therefore, in-depth studies of aging are particularly important.

Bioactive peptides are short peptides consisting of 2–20 amino acid residues. They have positive effects on body functions and generally have antibacterial, antihypertensive, antioxidant, and anti-inflammatory effects [8]. Natural bioactive peptides can be generally divided into two categories: endogenous peptides, which are naturally released from precursor proteins and secreted from cells, and exogenous peptides, which are produced by enzymatic hydrolysis of proteins or by biosynthesis or organic synthesis [9,10]. Bioactive peptide resources have been found in plants (soybeans, walnuts, rice bran, etc.), animals (some fish, dairy products, etc.), and some fungi and bacteria (yeast, lactic acid bacteria, etc.). The bioactive peptides used in early research were mainly derived from milk, cheese, and other dairy products. As research has progressed, active peptides have also been derived from other foods, including animal products as well as plant products [11]. They have been widely used in animal research, especially in rodents, but with limited research in humans. This is because rodents are easy to breed in the laboratory setting, have a short life cycle, and can be rapidly bred. Rodents also share similar genes and physiological functions with humans, making them ideal experimental animal models [12,13]. In this paper, we review the recent progress in anti-aging research involving the use of bioactive peptides in animal models, especially in rodents. In addition, we also highlight that aromatic residue, such as Trp, in some of the reported active peptides, can confer their anti-inflammatory and antioxidant activity. Moreover, many studies on active peptides mainly focus on the direct effects of these exogenous active peptides but ignore their indirect effects through regulating endogenous antioxidants in vivo. For example, exogenous active peptides can enhance endogenous antioxidative activity by increasing the levels of glutathione (GSH), superoxide dismutase (SOD), and bone-derived neurotrophic factor (BDNF) [14,15,16]. 

We divide this review into several sections based on the anti-aging effects on different organs. In each section, we review the mechanism of aging and the mechanism of action for the anti-aging effect of these bioactive peptides in each organ. We also summarize their common mechanism of action in different organs and the synergistic regulatory effects between endogenous and exogenous active peptides.

## 2. Bioactive Peptides Delay Skin Aging

### 2.1. Skin Aging

Skin is the largest organ and the body’s first barrier of defense against external pathogens. The skin protects the body from environmental damage and invasion of pathogens, and it is responsible for managing body temperature, sensation, and secretion function. Aging can cause different degrees of skin damage and interfere with the normal physiological function of other organs in the body [17]. The etiology of skin aging includes many factors. Among them, internal aging and photoaging are most common. Aging can alter the structure, function, and appearance of the skin, eventually leading to the increase of wrinkles, loss of elasticity, sagging, and pigment precipitation [18]. The main mechanisms of skin aging are the decrease of antioxidants in the skin, inflammation, and the degradation of collagen by increased MMPs [19]. Anti-aging bioactive peptides often act on these aging mechanisms. For example, oral collagen hydrolysates (CHs) can inhibit the activity of MMPs to reduce the degradation of collagen fibers [20]. Active peptides can reduce skin photoaging by scavenging free radicals [21]. Some bioactive peptides can reduce inflammation. In general, both endogenous and exogenous active peptides can down-regulate the factors causing skin aging. We discuss these in detail below.

### 2.2. Antioxidant Peptides in Delaying Skin Aging

**Figure 1:**
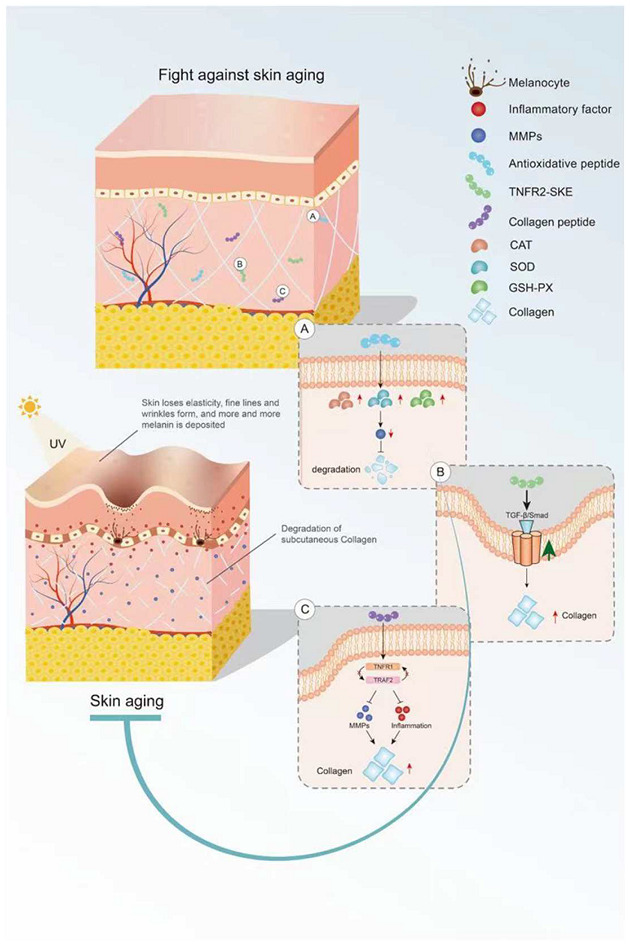
Mechanism of bioactive peptides in delaying skin aging. (**A**) Antioxidant peptides can increase the activity of antioxidant enzymes. (**B**) Bioactive peptides retard skin aging through the TGF-β/Smad pathway. (**C**) Active peptides inhibit inflammation and MMP activity. This figure cannot be reproduced without author permission.

Bioactive peptides derived from some animal proteins have antioxidant activity. These bioactive peptides can delay skin aging by regulating oxidative stress (Figure 1). For example, the collagen peptide extracted from the swim bladder of Sturgeon can increase the activities of catalase (CAT), SOD, and GSH peroxidase (GSH-PX) and decrease the activity of MMPs in skin tissue from Sprague-Dawley rats, as well as reduce the degradation of collagen by MMPs [22]. In recent years, some insect proteins with biological activity have also been found. For example, Eupolyphaga sinensis walker polypeptides (EPs) is a polypeptide mixture with a molecular weight of less than 3.3 kDa obtained from enzymatic digestion that can significantly improve the activity of antioxidant enzymes and reduce the generation of harmful free radicals. Thus, EPs can reduce the UV-irradiation-induced increase in epidermal thickness and elastic fiber breakage and restore the content of collagen [23]. In both cases, the mechanism of action of these exogenous active peptides is mainly to improve the activity of antioxidant enzymes in the skin and reduce the activity of MMPs and the degradation of collagen.

### 2.3. Anti-Inflammatory Peptides in Delaying Skin Aging

Some endogenous peptides with anti-inflammatory effects have been used to delay skin aging. The tripeptide TNFR2-SKE (362.4 Da) derived from the tetrapeptide of TNF receptor-associated factor 2 (TNFR2) showed a good protective effect against skin photoaging. TNFR2-SKE can block the interaction between TNFR1 and TRAF2 and inhibit the inflammation induced by TNF-2 (Figure 1). Intraperitoneal administration of TNFR2-SKE to UVB-irradiated six-week-old male DBA/2 mice was shown to significantly improve epidermal thickness and pigment cell proliferation [24]. MOTS-C is a 16-peptide from the MDP family derived from mitochondria with a molecular weight of 2174.61 Da. This bioactive peptide can regulate cell metabolism and inflammation [25,26]. In a D galactose-induced aging mouse model, treatment with MOTS-c was shown to increase collagen fiber content in the dermis by increasing NRF2 and MFN2 and decreasing interleukin-6 (IL-6). The anti-aging activity of MOTS-c is likely achieved by reducing inflammation [27]. Thus, both TNFR2-SKE and MOTS-C active peptides showed good performance in significantly alleviating skin inflammation and increasing collagen fiber content in mice. These endogenous active peptides can delay skin aging through their anti-inflammatory effects. However, there are many endogenous anti-inflammatory polypeptides in the body, and their anti-aging effects on the skin remain to be explored.

### 2.4. Peptides in Reducing Collagen Hydrolysis

Collagen is the main component of the dermis, and its content decreases with age. Skin sagging and wrinkles are caused by a decrease in collagen content. It is noteworthy that oral CHs can reduce skin laxity and wrinkles [28] and delay skin aging. Fish skin and fish scales are generally rich in collagen. Two collagen hydrolysates (ACH and CCH) prepared from fish skin can up-regulate the transforming growth factor β (TGF-β)/Smad signaling pathway related to collagen synthesis and increase the amount of collagen. CHs have a good protective effect on skin laxity, as shown in 13-month-old female KM mice [29]. Collagen hydrolysate CPNS (Gly-Pro and Pro-Hyp) [30] and CP [31] prepared from fish scales can significantly attenuate the increase in epidermal thickness and water loss and the decrease in dermal hyaluronic acid (HA) induced by UVB irradiation, as well as recover HA loss by regulating hyaluronan synthases 1 (HAS1), hyaluronan synthases 2 (HAS2), and hyaluronidase 2 (HYAL2). Another elastin hydrolysate (EH) prepared from the bovine artery is composed of four polypeptides: Gly-Leu-Pro-Tyr (GLPY), Pro-Tyr (PY), Gly-Leu-Gly-Pro-Gly-Val-Gly (GLGPGVG), and Gly-Pro-Gly-Gly-Val-Gly-Ala- Leu (GPGGVGAL). EH can inhibit UV-induced skin thickening and sebaceous gland hyperplasia in mice and promote moisturizing of the skin. GLPY and GPGGVGAL have better inhibitory effects on elastase and thus can reduce extracellular matrix (ECM) degradation and improve the activity of UV damaged fibroblasts [32]. Collagen hydrolysis is the main cause of skin sagging, and the supplement of some collagen hydrolytic peptides can reduce the hydrolysis of collagen by MMPs. However, the detailed underlying mechanism is still unclear and needs to be further explored.

## 3. Bioactive Peptides and Brain Aging

### 3.1. Brain Aging

In the process of aging, brain function will gradually decline, which is manifested by a decline of learning ability and memory, as well as attention, decision-making ability, sensory perception, and motor ability. The prevalence of some neurodegenerative diseases, such as AD, Parkinson’s disease (PD), and stroke, also increases with age. The development of these diseases is related to mitochondrial dysfunction, accumulation of oxidative damage, and increased inflammation [33]. AD is the most common neurodegenerative disease. Currently, abnormal folding of Aβ1-42 produced by the metabolism of amyloid precursor protein (APP) is considered to be the main cause of AD pathology [34]. Iron is involved in many biological processes in the brain and plays an important role in maintaining normal brain function. However, an iron imbalance can cause toxic effects on the brain. When the iron concentration is too high, it can increase the misfolding of Aβ and promote the development of AD [35]. The role of oxidative stress and inflammation in the development of AD is well known, and some new therapeutic targets have become research hotspots. Serotonin receptors (5-HT4R) have been found to reduce Aβ production. Many 5-HT4R agonists have been studied, but their potential therapeutic effect on AD has rarely been studied in vivo [36]. Glycosylation of proteins produces advanced glycation end products (AGEs) that can cause neurodegeneration. When glyoxalase activity is reduced, the ability of these toxic glycosylated proteins to be eliminated is significantly reduced, leading to neurological disease [37]. The relationship between the gut microbiome and aging and the development of AD has been confirmed, but no clear mechanism has been elucidated. In a recent report, we found that intestinal dysregulation of Firmicutes and Bacteroidetes promotes T helper 1 (Th1) cell infiltration and promotes microglia differentiation in a pro-inflammatory direction. This may be related to the development of AD [38]. Bioactive peptides can exert their anti-aging effect on the brain through various mechanisms. They can increase antioxidant enzyme activity, reduce inflammation, increase the removal ability of iron and AGEs, increase expression of 5-HT receptors, and regulate the gut microbiota.

### 3.2. Antioxidant Peptides in Delaying Brain Aging

**Figure 2:**
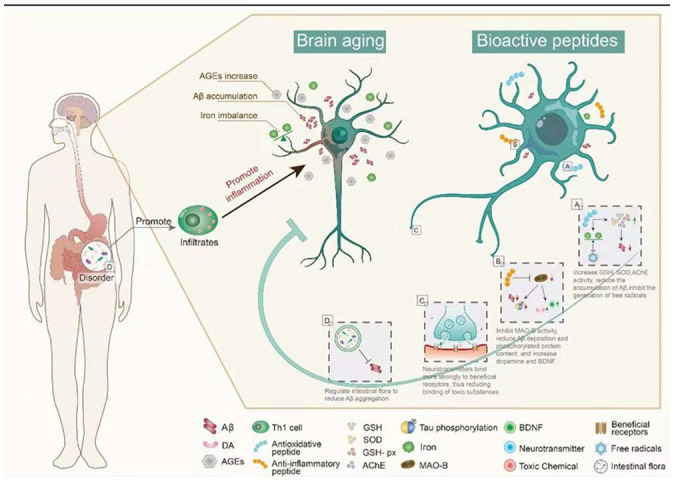
The main mechanism of bioactive peptides in delaying brain aging. (**A**) Bioactive peptides reduce Aβ accumulation by regulating oxidative stress. (**B**) The bioactive peptides inhibit the activity of MAO-B, up-regulate BDNF, and reduce the aggregation of Aβ. (**C**) Bioactive peptides reduce brain damage caused by toxic substances in the brain. (**D**) Bioactive peptides reduce Aβ aggregation by regulating intestinal microbiota. This figure cannot be reproduced without author permission.

Carnosine (CAR) is an endogenous dipeptide (β-Ala-L-His) existing in muscle, blood, and the brain. CAR has good antioxidant activity and can attenuate neurological diseases caused by aging; CAR supplementation reduces the accumulation of Aβ in the hypothalamus and prefrontal cortex of aging rats and has potential therapeutic effects on AD [39]. After CAR treatment, GSH levels and SOD and GSH-Px activity were increased, whereas acetylcholinesterase (AChE) activity was significantly decreased (Figure 2), and there was a significant reduction in neuronal apoptosis, brain edema, and inflammation in D-galactose treated rats [40]. With aging, iron gradually accumulates and induces the generation of free radicals, promoting the formation of Tau and Aβ oligomers, which are neurotoxic and the main cause of AD [41]. The amount of iron found in the brains of AD patients is much higher than that of normal brains, suggesting that excess iron may be one of the causes of AD [35,42]. To better understand the effects of iron, researchers have synthesized the peptides with the ability to remove iron ions. Pentapeptide YHEDA (Tyr-His-Glu-Asp-Ala) and polypeptide mixture HAYED (5) Five (His-Ala-Tyr-Glu-Asp) repeat sequences are two synthetic active peptides with good iron ion scavenging ability (Figure 2). They can prevent the decrease of blood oxygen metabolism, inhibit the generation of free radicals, and reduce the damage in brain tissue, effectively improving cognitive impairment in senescent (SN) mice (25 months old) [43,44]. However, many high-quality natural antioxidant peptides have yet to be discovered and utilized in anti-aging studies. For example, many plant-derived bioactive peptides have antioxidant activities, and the research and development of these active peptides in aging studies will be of great significance in delaying brain aging [45].

### 3.3. Anti-Inflammatory Peptide in Delaying Brain Aging 

Synthetic bioactive peptides are being increasingly produced for the treatment of different diseases. Liraglutide, a synthetic long-acting glucagon-like peptide 1 (GLP-1) analog, is widely used in the treatment of diabetes mellitus and CVDs. Recently, it has been speculated that liraglutide may have neuroprotective effects [46,47]. In senescence accelerated mouse P8 (SAMP8) mice (model of AD-like dementia), liraglutide treatment can improve spatial long-term memory and increase the number of hippocampal neurons [48,49]. The active peptides in dairy products have been long known, and there have been some reports that these active peptides can delay brain aging, mainly with the improvement of AD symptoms. The Whey protein hydrolysate tryptophan-methionine and tryptophan-tyrosine, extracted from fermented dairy products, can improve the cognitive impairment of AD mice. Inflammation and Aβ_1-42_ deposition in the cerebral cortex and hippocampus are also significantly reduced in 5 × FAD transgenic mice fed tryptophan-tyrosine [50]. Notably, Tryptophan-Tyrosine dipeptide and whey protein hydrolysate GTWY (Gly-Thr-Trp-Tyr) can increase dopamine (DA) content in the hippocampus and frontal cortex of AD mice by inhibiting the activity of monoamine oxidase B (MAO-B) [51,52,53]. β-lactolin, an active polypeptide extracted from whey protein hydrolysate, has been shown to improve cognitive impairment. Specifically, β-lactolin can reduce amyloid plaque deposition and phosphorylated Tau protein content in the cerebral cortex of 5 × FAD transgenic mice (AD mice), as well as increase DA and BDNF levels, thereby improving the cognitive impairment [54]. BDNF is one of the most widely distributed neurotrophic factors in the brain, and it plays an important role in regulating synaptic growth, neuroprotection, and affecting memory and cognition in vivo [55]. β-lactolin can increase the expression of BDNF in vivo (Figure 2). This is an example of how exogenous active peptides have a regulatory effect on endogenous active substances. Thus, exogenous active peptides not only play a therapeutic role in some antioxidant and anti-inflammatory pathways but also enhance the expression of endogenous active peptides to treat some diseases. The mechanism of action of exogenous active peptides may differ from endogenous ones, but they can supplement the body’s defense system.

### 3.4. Regulation of Peptide Receptors in Delaying Brain Aging

Serotonin (5-HT) is an important neurotransmitter that is involved in a variety of brain activities and functions. 5-HT receptors decrease gradually in the aging process. Serotonergic neurons are widely distributed in the brain. Reduction of 5-HT receptors can cause functional impairment of these neurons and lead to cognitive impairment. CAR is a dipeptide extracted from the meat. It can enhance 5-HT binding to its receptor and restore the regional senage-induced decrease in serotonin to normal levels [56,57]. Pituitary adenylate cyclase activated polypeptide (PACAP) is an endogenous active polypeptide with 38 amino acid residues and has a neuroprotective effect. It is widely distributed in the brain, pancreas, gonad, and respiratory tract. PACAP38 can be cleaved to form a 27 amino acid polypeptide, PACAP27 [58]. The level of PACAP gradually decreases in the normal aging process, and decreased PACAP levels have been found in the brain tissues of AD patients [59]. PACAP27 and PACAP38 can reduce the accumulation of Aβ in the brain by activating pituitary adenylate cyclase-activating polypeptide (PAC1), which causes the shedding of the receptor for advanced glycation end products (RAGE) of late glycation end products on the cell surface [60]. In summary, these peptides act on receptors, promoting the binding of beneficial receptors in neurons but reducing the binding of toxic substances.

### 3.5. Intestinal Microbiota Regulation by Peptides in Delaying Brain Aging

The link between the gut microbiota and AD is widely recognized, and many substances, including bioactive peptides, have been reported to regulate the gut microbiota. Some active peptides can regulate the intestinal microbiota in a beneficial direction by reducing Aβ aggregation, which has a potential role in the treatment of AD by regulating the intestinal microbiota [61]. The walnut protein hydrolysate PW5 (Pro-Pro-Lys-Asn-Trp) identified from walnut protein can reduce Aβ aggregation and improve cognitive impairment in mice by regulating intestinal microbiota (Figure 2). PW5 fed to APP/PS mice (AD mice) can increase firmicutes in the intestinal microbiota, which may be associated with reduced Aβ aggregation in mice [62]. The association between intestinal microbiota and AD has been widely recognized, and many bioactive peptides have been used to regulate intestinal microbiota to improve AD symptoms, but the mechanism is still not deeply studied, and further exploration is needed.

## 4. Bioactive Peptides and Aging in Other Organs

Aging is an irreversible biological process. Organs in the body cannot avoid aging. This leads to a variety of chronic diseases, including CVD, chronic obstructive pulmonary disease (COPD), intermittent lung disease, and asthma [63,64]. The aging processes of these important organs are correlated, and complications of one organ often lead to multi-organ disease. For example, lung aging causes COPD, which causes systemic inflammation and increases the risk of non-alcoholic liver disease. Moreover, people with non-alcoholic liver disease are more likely to have chronic kidney disease (CKD) and CVD. Oxidative stress and inflammation play an important role in the pathogenesis of these diseases. Many bioactive peptides with antioxidant and anti-inflammatory activities have been used in the prevention and treatment of these diseases. However, the role of a peptide in a disease is often limited, and there is still a lack of research on the complications of these diseases.

### 4.1. Lung Aging

COPD is a major form of lung disease characterized by chronic inflammation of the windpipe. Aging and smoking are the main causes of COPD. People over the age of 65 are five times more likely to develop the disease than younger people [65,66]. COPD is often associated with metabolic abnormalities, CVD, skeletal muscle atrophy, and other chronic diseases. In the later stages of COPD, arteriosclerosis, oxidative stress, and inflammation are the main mechanisms of its progression. Persistent inflammation disrupts the normal function of the lungs and is one of the causes of other complications [67]. Other researchers point to systemic inflammation from COPD as a major cause of non-alcoholic fatty liver disease (NAFLD) [68].

#### 4.1.1. Antioxidant Peptides in Delaying Lung Aging

The human body is rich in peptides that play various biological activities in the body to adapt to different needs. The tripeptide GHK (glycyl-L-histidyl-L-lysine) is an active peptide existing in the human body, which has a high affinity for copper and can form a GHK-Cu complex. GHK-Cu has anti-inflammatory and antioxidant functions and can promote blood vessel growth and increase neural nutrition [69]. GHK-Cu has been shown to improve the symptoms of acute lung injury (ALI), which is usually accompanied by severe oxidative stress and inflammation. In ALI mice treated with GHK-Cu, SOD activity and GSH levels were significantly increased, and the NF-κB signaling pathway was blocked to reduce the release of inflammatory factors [70]. GHK-Cu is also a potential drug candidate for treating some chronic lung diseases such as COPD, asthma, and lung cancer [71].

#### 4.1.2. Anti-Inflammatory Peptide in Delaying Lung Aging

Since many plants are rich in active substances, they are widely studied for use in drug development. The cyclic peptide CPE extracted from hydrolysates of Pseudostellariae can effectively relieve the symptoms of COPD. CPE treatment can significantly reduce the degree of alveolar destruction and lung inflammation, increase alveolar space, and regulate various cytokines. CPE treatment also reduces several mRNAs for TLR4, the adaptor protein MyD88 and activator protein-1 (AP-1), and active phosphorylated forms of proteins (P-JNK, P-P38, and P-TAK1) in alveolar macrophages in a COPD rat model [72]. These results suggest that CPE can act on the TLR4-MyD88-JNK/P38 signaling pathway and inhibit the release of important inflammatory factors to reduce lung inflammation. Thus, CPE has therapeutic potential for treating COPD. As an exogenous active peptide, CPE has a similar mechanism of action as GHK-Cu; both can block inflammatory pathways and reduce the release of inflammatory factors to attenuate lung inflammation. However, it would be interesting to explore whether CPE plays a synergistic role with GHK-Cu in vivo.

### 4.2. Liver Aging

A high-fat diet can cause NAFLD and non-alcoholic hepatitis (NASH), which is one of the major causes of cirrhosis and hepatocellular carcinoma (HCC). According to research, older people are more likely to develop NAFLD [73]. The liver is an important organ in the body. Dysfunction of antioxidant enzymes can reduce the ability of liver cells to remove peroxides, leading to the damage of mitochondrial DNA and mitochondrial dysfunction, resulting in liver aging [74,75]. Changes in the gut microbiome can also cause liver disease. For example, chronic inflammation, known as “metabolic inflammation”, caused by changes in the microbial metabolites of the gut microbiome, can lead to NAFLD. Analysis of these altered gut microbes has revealed a significant increase in Proteobacteria, a group of microbes that may be responsible for NAFLD [76]. NAFLD, in turn, can increase the risk of atherosclerosis and accelerate the development of atherosclerosis symptoms. This is supported by a correlation in lesions of several organs [77]. Internal organs also interfere with each other as the body ages. This is exemplified by the fact that the severity of NAFLD increases the risk and severity of CKD [78].

#### 4.2.1. Antioxidant and Anti-Inflammatory Peptides in Delaying Liver Aging

The body secretes some active polypeptides when maintaining normal physiological functions. Adropin is a peptide hormone that is expressed in the liver and can regulate blood glucose and lipid homeostasis. Studies have shown that adropin knockout can increase liver inflammation, liver steatosis, and fibrosis in mice, promoting the development of NASH. The underlying changes due to the knockout, such as reduction of Nrf2 transcriptional activity, GSH level, and mitochondrial membrane potential, can be normalized by adropin supplementation. These results indicate that adropin can delay the development of NASH by maintaining mitochondrial homeostasis and increasing antioxidant enzyme activity [79]. This is an example of how the liver can secrete beneficial active peptides to maintain normal physiological function. However, in the aging process, when the liver has reduced ability to produce such peptides, there is subsequent liver damage. Thus, the supplementation of these active peptides in an aging individual can maintain the normal function of the liver and prevent liver damage. It is worth mentioning that after long-term research on the anti-aging of active peptides, our research group has found that the mice treated with the active peptide derived from rice bran have reduced aging characteristics caused by galactose. This peptide is extracted from rice bran protein hydrolysate, named KF-8 (Lys-His-Asn-Arg-Gly-Asp-Glu-Phe), can reduce oxidative stress in D-gal-treated mouse livers by inhibiting the NF-κB/p38 signal transduction pathway and delaying liver aging [80]. This suggests that some exogenous active peptides with antioxidant activity could be added to the diet as anti-aging supplements.

#### 4.2.2. Intestinal Microbiota Regulation by Peptides in Delaying Liver Aging

Liraglutide (discussed above) can improve the symptoms of NAFLD by regulating the gut microbiome. Specifically, liraglutide treatment can reduce Proteobacteria, a common factor in many diseases, and increase Verucommicrobia, which contributes to intestinal health and glucose homeostasis in the intestines of obese mice. Changes in the abundance of gut microbiota by liraglutide are associated with improvement of NAFLD symptoms and a reduction in inflammatory cell infiltration in the cecum and liver [81]. Mechanistically, liraglutide regulates the gut microbiome to reduce liver inflammation. There are many other bioactive peptides that can regulate the intestinal microbiome; however, the detailed mechanisms of their effects have not yet been elucidated.

### 4.3. Kidney Aging

The physiological function of the kidneys gradually deteriorates with aging, causing some kidney diseases [82]. With the increase of age, kidneys are also more vulnerable to oxidative damage, especially in the mitochondria of the kidney cells. Impaired mitochondrial function and cellular metabolism eventually lead to chronic renal failure [83]. CKD is an important cause of CVD because it can lead to high blood pressure and a decrease in the capillary density of the cardiac tissue. In addition, CKD reduces nitric oxide synthase expression in the vascular endothelium and increases renin–angiotensin system activity, resulting in increased release of superoxide and inflammatory cytokines and subsequent CVD [84].

#### 4.3.1. Antioxidant Peptides in Delaying Renal Aging

In the aging process, increased oxidative stress and chronic inflammation can lead to some kidney diseases. Most of the endogenous active peptides in the endocrine system can reduce oxidative stress and inflammation, and thus, these peptides have potential therapeutic for alleviating kidney diseases. Mitochondrial targeted peptide SBT-20 (also known as SS-20) is a synthetic tetrapeptide that can reduce ROS and maintain the normal production of the electron transport chain and ATP. SBT-20 can reduce the expression of mitochondrial mitotic protein Drp1 and increase the expression of mitochondrial fusion protein 2 (Mfn2) to maintain the normal structure and function of mitochondria. SBT-20 treatment can decrease the expression of inflammatory cytokines IL-1β, IL-6, NF-κB1, and NF-κB2 in the kidney and alleviate the symptoms of chronic renal failure (CRF) in CRF mice [85]. Compared with endogenous active peptides, SBT-20 also has regulatory effects on inflammation and oxidative stress. With the technological development of peptide synthesis in vitro, the synthesis of these active peptides to meet the therapeutic needs of different diseases would be a powerful and desirable approach in the future.

#### 4.3.2. Anti-Inflammatory Peptides in Delaying Renal Aging

Compared with endogenous active peptides produced in animals, exogenous active peptides from plants can also reduce the symptoms of CRF through similar mechanisms. Soy is rich in proteins that can be hydrolyzed into some bioactive peptides. The soybean protein hydrolysate (SPH) can lower blood pressure and maintain normal renal function. We showed that feeding rats (5/6 nephrectomized model) SPH can reduce ACE activity and TNF-α levels. These results suggest that SPH can reduce renal inflammation in CRF by down-regulating TNF-α activity [86]. Thus, exogenous active peptides are similar to endogenous active peptides in slowing CRF progression. Since exogenous active peptides are more widely derived, they hold great promise for providing therapeutic effects for many diseases.

### 4.4. Aging of the Heart and Blood Vessels

Complications of many diseases can increase the risk of CVD, and CVD can also promote lesions in other organs. The impaired endothelial cell function that is associated with aging can lead to vascular dilation and decreased anti-thrombotic ability, and eventually CVD. The main causes of CVD are activation of inflammatory signals induced by NF-κB, increases in MMP-9, and changes in TGF-β [87,88]. In addition, increased inflammation with aging can induce the expression of vascular endothelial growth factor (VEGF) family proteins. Although VEGF plays a beneficial role in some CVDs, over-expression of VEGF promotes the formation of new blood vessels, which in turn contributes to the development of atherosclerotic pathology [89].

#### 4.4.1. Antioxidant Peptides in Delaying Cardiovascular Aging

Some active peptides extracted from animals and plants have potential therapeutic effects on CVD. For example, some exogenous active peptides extracted from grains may improve CVD [90]. Rice α-globulin hydrolysate Try-Try-Gly-Gly-Glu-Ser-Ser-Ser-Glu-Gln-Gly (YYGGESSSEQG) and Ser-Glu-Ser-Glu-Met (SESEM) extracted from rice can improve the symptoms of atherosclerosis in mice. They can down-regulate the TNF-α pathway and NF-κB in the aorta and aortic root tissues and reduce oxidative stress levels and inflammatory factors in apolipoprotein E-deficient mice [91]. Another exogenous active peptide, zebra blenny protein hydrolysates (ZBPHs), extracted from zebrafish protein, has a good antioxidant effect and can reduce the lipid deposition and apoptosis of cardiac cells induced by a high cholesterol diet in hypercholesterolemic rats. This is a potential therapeutic agent for CVD [92]. Since these two exogenous active peptides are isolated from rice and zebrafish proteins, respectively, they are widely available and can easily be incorporated into the diet.

#### 4.4.2. Anti-Inflammatory Peptide in Delaying Cardiovascular Aging

In normal conditions, the heart can secrete some active peptides to maintain the physiological functions of the cardiovascular system. Natriuretic peptide (NP) is a kind of polypeptide that can maintain normal function of the heart, blood vessels, and kidney, and is associated with some CVDs [93]. NPs mainly exist in two forms: atrial na-triuretic peptide (ANP) and cerebral natriuretic peptide (BNP). BNP has been reported to treat myocardial infarction in mice. BNP can promote endothelial cell proliferation and myocardial vascularization in the infarcted and non-infarcted areas in the hearts of mice following myocardial infarction. The action of BNP is mediated by P38 MAP kinase [94]. Adropin, a peptide used to treat the non-alcoholic liver disease, can also be used to treat CVDs. It can reduce inflammation and migration of vascular smooth muscle cells, improve symptoms, and reduce intravascular plaque significantly [95]. Since adropin can be used to treat liver aging and atherosclerosis, it would have an added therapeutic potential to treat the cardiovascular complications of NASH. 

## 5. Conclusions

Continuous improvement of biomedical research and healthcare has resulted in a significant increase in life span and the aging population. However, this has created a subsequent problem because aging is associated with many diseases. Therefore, great efforts have been made in anti-aging research, and many bioactive peptides have been discovered to have anti-aging activity. Bioactive peptides can be endogenously produced in the body, but more and more bioactive peptides are being exogenously produced from natural products or biosynthesis. The mechanisms of action of these bioactive peptides mainly involve their antioxidant and anti-inflammatory activities. Interestingly, some exogenous and endogenous active peptides have synergistic effects. Several organs in the body share similar aging mechanisms, and one organ disease can affect multiple organs. Thus, bioactive peptides can have anti-aging effects on multiple organs.

Although bioactive peptides have been widely used in anti-aging studies of rodents, it is not clear whether these active peptides exert their anti-aging effects in humans through similar mechanisms. Therefore, anti-aging research using genetically similar animal models, such as primates, is needed before many bioactive peptides can be tested in human clinical trials. 

Here, we have reviewed the anti-aging activity of bioactive peptides, most of them from the hydrolysate of some food, as well as some synthetic active peptides and endogenous active peptides. These peptides were ingested by mouth, gavage, and injection in rodent models, and behavioral and physiological changes in these animals demonstrated the protective benefits of these peptides (reduced disease symptoms). It is worth mentioning that these active peptides have no adverse side effects and toxicity to experimental animals within the range of experimental concentrations, which also indicates that bioactive peptides are non-toxic and hypoallergenic active substances.

In this paper, we have introduced some of the anti-aging activities of active peptides, but there is additional research in the literature on other peptides. In view of this, Table 1 provides more information on other active peptides that are not discussed in detail in the text, as well as summarizing the animal models used in the studies of these active peptides and methods and dosages of peptide administration for reference of interested scholars. In addition, the main abbreviations that appear in this article are given in Table A1 of the Appendix A.

## Figures and Tables

**Table 1:** Bioactive peptides with anti-aging activity.

Classification	Name and Delivery Way	Source	Rodent Model	Target Organ	Mechanism
Food-derived active peptide	Walnut protein hydrolysates(WPH) Oral gavage for 21 days Low: 333 mg/kg High: 666 mg/kg	Walnut	Alzheimer’s disease model mice aged 6–8 weeks scopolamine solution (1.0 mg/kg)	Brain	SOD↑ GSH-Px↑ CAT↑ Nrf2↑ BDNF↑ CREB↑ MDA↓ TNFα↓ AchE↓ Trp-, Tyr-, or Phe-containing peptide has high affinity to Keap1 and Ache, so it can increase the activity of NRF2 and reduce the activity of Ache, which ultimately increases antioxidant capacity and anti-inflammatory ability and leads to increased BDNF, CREB transcription [15]
	Walnut protein hydrolysate and its low-molecular-weight fraction (WPH/WPHL) Oral gavage for 21 daysWPH: 666 mg/kgWPHL: 666 mg/kg	Walnut	Alzheimer’s disease model mice aged 6–8 weeks LPS (300 μg/kg bw)	Brain	SOD↑ GSH-Px↑ CAT↑ MDA↓ TNFα↓ TNFα↓ IL-6↓ IL-1β↓ Trp, Gly, Leu residues, hydrophobic amino acids, and aromatic amino acids in polypeptides can inhibit the expression of pro-inflammatory factors TNF-α, IL-1β, and IL-6 and reduce inflammation [16]
	Tyr-Val-Leu-Leu-Pro-Ser-Pro-Ly (walnut protein hydrolysates) Continuous injection for 4 weeks 60 mg/kg bw	Walnut	Alzheimer’s disease model mice (C57BL/6) 5–6 week oldscopolamine solution (1 mg/kg bw)	Brain	ATP↑ PINK1↑ Parkin↑ NRF2↑ LC3 II/LC3 I↑ Beclin↑ KEAP1↓ p62↓ It can increase antioxidant capacity through Nrf2 signaling pathway and increase the expression of Beclin-1, Parkin, and PINK1 to enhance mitochondrial autophagy capacity [96]
	Alcalase potato-protein hydrolysates (IF) Oral administration 3 weeks 1 mg/kg bw	Potato	Senescence-Accelerated mice (SAMP8) 6 months high-fat diet	Liver/heart	pAKT↑ Sirt1↑ pAMPK↑ PGC1α↑ pFOXO3a↑ Bax↓ GOT↓ GPT↓ LDL↓ ANP↓ BNP↓ pGATA4↓ It can down-regulate cardiac hypertrophy markers ANP and BNP, reduce inflammation in the heart and liver, and reduce apoptosis by stimulating the activity of Sirt1 [97]
	Alcalase potato protein hydrolysate (APPH) Oral administration 4 weeks Low: 15 mg/kg/day Middle: 45 mg/kg/day High: 75 mg/kg/day	Potato	Sprague-Dawley (SD) rat 23 months old high-fat diet	Heart	p-p38/p38↓ GSN↓ p-Gata4↓ TGFβ↓ APPH has good lipid solubility and can reduce myocardial hypertrophy and fibrosis in aging rats through TGF-β/GSN pathway [98]
	Casein hydrolysates Continuous injection 10 weeks 200 mg/kg	Casein	Diabetic rat high-fat diet	Liver	NRF2↑ HO-1↑ SOD↑ GSH↑ MDA↓ By enhancing Nrf2 translation, the activity of antioxidant enzymes was enhanced, and the activities of DPP-IV and ACE were inhibited, among which dipeptide WM could inhibit Keap1/Nrf2 interaction [99]
	Wheat germ albumin hydrolysates ((Ala-Asp-Trp-Gly-Gly-Pro-Leu-Pro-His)) Continuous injection 1 week 4 mg/kg	Wheat	Diabetic mice 6 weeks old	Vascular	pAMPK/AMP↑ pPKCζ/PKCζ↓ NOX4↓ ROS↓ pAKT/AKT↓ Inhibition of NOX4 expression through the PKCζ/AMPK signaling pathway reduced oxidative stress levels and the release of inflammatory factors [100]
	Collagen hydrolysate Pro-Hyp Oral administration 4 weeks 210 mg/kg	Porcine skin	Chronic kidney disease mice 6 weeks old	Kidney	Liver iron content↑ EPO↑ HIF-2α↑ Hepcidin↓ TNF-α↓ IL-1β↓ IL-6↓ NF-κB↓ COX2↓ It reduces inflammation by regulating inflammatory pathways and plays a protective role in regulating HIF-2α, EPO, and Hepcidin [101]
	Anchovy hydrolysates Pro-Ala-Tyr-Cys-Ser (PAYCS) 20 days 0.2 mM/kg/day	Anchovy	Alzheimer’s disease model mice 6 weeks old Scopolamine solution (1 mg/kg bw)	Brain	Ach↑ AchR↑ Nrf2↑ BDNF↑ SOD↑ The antioxidative effects of PAYCS and PAY may be related to the Try active phenolic structure in the sequence and the hydrogen donor of the sulfhydryl group in Cys. Both active peptides have the ability to promote the binding of Ach and AchR [102]
	Soy protein isolate (SPI) Oral administration 8 weeks	Soy	Obese rat 6 weeks old	Liver	NPTX2↑ GPT↑ INMT↑ HAL↑ The increased expression of NPTX2 reduced the inflammation of the rat liver, the increased expression of GPT may be related to mitochondrial energy metabolism, the increased expression of INMT may be related to the relief of NAFLD symptoms, and the increased expression of HLT can consume excess protein in the liver [103]
	Walnut protein hydrolysate Oral administration Low: 0.32 g/L Middle: 0.96 g/L High: 2.88 g/L	Walnut	Skin-aging model rat Exposed to UV-R	Skin	Elastin↑ Fibrillin-1↑ MMP-1↓ Increasing the expression of Col I, Col III, HYP, and HA and significantly attenuated the activity of MMP-1 [104]
	Eucheuma hydrolysate (EZY-1) 28 day 0.25 mg/kg 0.5 mg/kg 1 mg/kg 50 mg/kg	Eucheuma	Pulmonary fibrosis mice (C57BL/6J) 8 weeks old injected with 3.5 mg/kg of bleomycin	Lung	T-SOD↑ GSH-Px↑ HYP↓ MDA↓ pSmad3↓ EZY- 1 is easily absorbed in the intestinal tract, and its hydrophobic point facilitates the entry of EZY-1 into cells, while EZY-1 can reduce pulmonary fibrosis through TGF-β/Samd signaling pathway. [105]
	Egg white protein hydrolysate (EWPs) Gavage 14 days Low: 50 mg/kg Middle: 100 mg/kg High: 200 mg/kg	Egg	Colitis model mice (BALB/c) administered 3% (*w*/*v*) DSS	Gut	Candidatus_Sacchar-imonas↑ norank_f_Ruminococcaceae↓ Ruminiclostridium↓ TNF-α↓ IL-6↓ IL-8↓ EWPs contain Trp, Try, His, and Met, which make it have good antioxidant activity and can reduce the release of inflammatory factors by increasing the content of Lactobacillus and Candidatus-Saccharimonas in the gut [106]
	Whey protein hydrolysate (WHP) Gavage 30 days Low: 0.3 g/kg Middle: 1.5 g/kg High: 3.0 g/kg	Egg	D-galactose-treated mice (C57BL/6N) 6 months 100 mg/kg	Brain	SOD↑ GSH-Px↑ AChE↑ p-CaMKII↑ MDA↓ TNF-α↓ IL-1β↓ TNF-α↓ WHP can reduce the release of inflammatory factors, increase the activity of antioxidant enzymes, and enhance the activities of AchE and P-CamKII, which play an important role in maintaining synaptic plasticity [107]
A peptide encrypted from the venom of Tityus serrulatus scorpion	Lys-Pro-Pro (KPP)	Scorpion	Mice 10 weeks old	Heart	pPLN/PLN↓ pERK/ERK↓ KPP regulates cellular stress-related proteins and exerts cardioprotective effects through PLN dephosphorylation [108]
Secretory bioactive peptide	Humanin (HNG) Injections 14 months 4 mg/kg	Mitochondria	Aging mice (C57BL/6N) 18 months	Heart	pAKT↑ pGSK3β↓ 4-HNE↓ TGF-β1↓ FGF-2↓ MMP-2↓ HNG down-regulated the expression of GSK-3β through Akt pathway, reduced myocardial apoptosis, down-regulated FGF-2 and MMP-2 expression, and inhibited cardiac fibrosis [109]
Peptide hormone	Melatonin Injections 30 days 10 mg/kg	Pineal gland	Aging mice 8 weeks old D-galactose 100 mg/kg	Brain	SNAP-25↑ PSD95↑ GluR1↑ p-CREB↑ ROS↓ GFAP↓ p-IKKβ↓ NF-κB↓ COX-2↓ NOS2↓ IL-1β↓ TNFα↓ p-JNK↓ Melatonin can reduce synaptic damage caused by oxidative stress and neuroinflammation through RAGE/NFκB/JNK pathway and has a good therapeutic effect on neurodegeneration [110]

Annotation: “↑” Up-regulation, “↓” Down-regulation, the abbreviations in Table 1 are all listed in Table A1.

## Data Availability

Data available in a publicly accessible repository (https://pubmed.ncbi.nlm.nih.gov, accessed on 21 January 2022).

## Appendix A

**Table A1:** The full name of abbreviations.

Abbreviations	Full Name	Abbreviations	Full Name
SOD	Superoxide dismutase	Samd	Drosophila mothers against decapentaplegic protein
GSH-Px	Glutathione peroxidase	pAMPK/AMP	AMP-activated protein kinase/AMP
GSH	Glutathione	pPKCζ/PKCζ	Anti-phospho-protein kinase ζ
CAT	Catalase	NOX4	Antibodies against NADPH oxidase4
Nrf2	Transcription factor nuclear factor erythroid 2-related factor 2	EPO	Erythropoietin
BDNF	Brain-derived neurotrophic factor	HIF-2α	Hypoxia-inducible factor
CREB	cAMP-response element-binding protein	NF-κB	Nuclear factor-kappa beta
AchE	Acetylcholinesterase	COX2	Cyclooxygenase
MDA	Malondialdehyde	Ach	Acetylcholine
TNFα	Tumour necrosis factor-α	AchR	Cetylcholine receptor
IL-8	Interleukin-8	NPTX2	Neuronal pentraxin 2
IL-6	Interleukin-6	INMT	Indolethylamine N-methyltransferas
IL-1β	Interleukin-1β	HAL	Histamine ammonia-lyase
ATP	Adenosine triphosphate	MMP	Atrix metalloproteinase
PINK1	Mutations in the PTEN-induced kinase 1	ERK	Extracellular signal-regulated kinase
Parkin	Parkin RBR E3 ubiquitin protein ligase	HYP	Hydroxyproline
LC3Ⅱ/LC3Ⅰ	Microtubule-associated protein light chain 3	p-CaMKII	Phosphorylated Ca2+/calmodulin-dependent protein kinase II
KEAP1	Kelch-like ECH-associated protein 1	PLN	Dephosphorylation of phospholamban
p62	Protein sequestosome 1/p62	ERK	Extracellular regulated protein kinases
pAKT	Phosphorylated protein kinase B	pGSK3β	Phosphorylated glycogen synthase kinase-3beta
Sirt1	Silencing information regulator 2 related enzyme	4-HNE	4-hydroxynonenal
pAMPK	Phosphorylated AMP-activated protein kinase	FGF-2	Fibroblast growth factor 2
PGC1α	Peroxisome proliferator-activated receptor-γ co-activator-1α	SNAP-25	Synaptosomal associated protein 25
pFOXO3a	Phospho forkhead box O3a	PSD95	Postsynaptic density proteins
GOT	Glutamic oxaloacetic transaminase	GluR1	Anti-phospho-AMPARs
GPT	Glutamic-pyruvic transaminase	p-CREB	Phosphorylated cAMP-response element-binding protein
LDL	Low-density lipoprotein	GFAP	Astrocytosis
ANP	Atrial natriuretic peptide	p-IKKβ	Phosphorylated IKKbeta
BNP	Cerebral natriuretic peptide	NOS2	Nitric oxide synthase-2
pGATA4	Phosphorylated GATA binding protein 4	p-JNK	Hospho-c-JunN-terminal Kinase
p-p38/p38	Phosphorylated p38 kinase/p38 kinase	TGFβ	Transforming growth factor-beta
GSN	Gelsolin		
